# Development of a Highly Sensitive β-Glucan Detection System Using Scanning Single-Molecule Counting Method

**DOI:** 10.3390/ijms22115977

**Published:** 2021-06-01

**Authors:** Yoshiyuki Adachi, Hidetaka Nakata, Tetsuya Tanabe, Daisuke Yamanaka, Takashi Kanno, Ken-ichi Ishibashi, Naohito Ohno

**Affiliations:** 1 Laboratory for Immunopharmacology of Microbial Products, School of Pharmacy, Tokyo University of Pharmacy and Life Sciences, 1432-1 Horinouchi, Hachioji, Tokyo 192-0392, Japan; ymnkd@toyaku.ac.jp (D.Y.); kannotak@toyaku.ac.jp (T.K.); ohnonao@toyaku.ac.jp (N.O.); 2 Biological Evaluation Technology, Olympus Corporation, 2-3 Kuboyama-cho, Hachioji, Tokyo 192-8512, Japan; hidetaka.nakata@olympus.com (H.N.); tetsuya.tanabe@olympus.com (T.T.); 3 Department of Host Defense and Responses, Kagawa Nutrition University, 3-9-21 Chiyoda, Sakado 350-0288, Japan; ishibashi.kenichi@eiyo.ac.jp

**Keywords:** (1→6)-β-glucanase, beta-glucan recognition protein, invasive fungal infection, *Limulus* amebocyte lysate assay, scanning single-molecule counting method

## Abstract

To overcome the limitations of the *Limulus* amebocyte lysate (LAL) assay method for the diagnosis of invasive fungal infection, we applied a reaction system combining recombinant β-glucan binding proteins and a scanning single-molecule counting (SSMC) method. A novel (1→3)-β-D-glucan recognition protein (S-BGRP) and a (1→6)-β-glucanase mutant protein were prepared and tested for the binding of (1→6)-branched (1→3)-β-D-glucan from fungi. S-BGRP and (1→6)-β-glucanase mutant proteins reacted with β-glucan from *Candida* and *Aspergillus* spp. Although LAL cross-reacted with plant-derived β-glucans, the new detection system using the SSMC method showed low sensitivity to plant (1→3)-β-D-glucan, which significantly improved the appearance of false positives, a recognized problem with the LAL method. Measurement of β-glucan levels by the SSMC method using recombinant β-glucan-binding proteins may be useful for the diagnosis of fungal infections. This study shows that this detection system could be a new alternative diagnostic method to the LAL method.

## 1. Introduction

As the number of immunocompromised patients increases, the number of opportunistic infections is also increasing annually [1]. The β-D-glucan (BG) test is a valuable diagnostic standard that covers a wide range of invasive fungal infections that have a poor prognosis [2]. Estimating the number for the year based on the data for June of the relevant year, the number of BG tests in Japan has rapidly increased from approximately 590,000 in 2010 to approximately 990,000 in 2018 [3]. Therefore, the medical value of the BG test is increasing. However, the current BG test method, the factor-G-based *Limulus* amebocyte lysate (LAL) assay, can produce false-positive reactions to 1,3-and 1,4-type BGs derived from cellulosic materials in the environment (e.g., gauze) [4,5,6].

Since the LAL assay reagent in BG testing detects both fungal and plant BGs [6], it is necessary to prevent contamination with plant BGs [7]. (1→3)-β-D-glucans are polysaccharides produced by plants, bacteria, and fungi. The chain length, degree of branching, and number and location of other glycosidic linkages, such as (1→4)-β- and/or (1→6)-β-bonds, vary widely. (1→3)-linked (1→4)-β-glucan structures are typically found in plant materials [8], but (1→3)- and (1→6)-β-D-glucosyl chains are more common in fungi [9]. (1→3)-β-D-glucans activate cells of the innate immune system by binding to glucan-specific receptors, such as dectin-1 and other cell membrane receptors [9]. Unlike mammals, insects have a unique pattern recognition receptor for (1→3)-β-D-glucan [9,10,11,12], called the BG recognition protein (BGRP). The interaction of BGRP with BG activates serine proteases, which in turn react with prophenoloxidase and phenol oxidase [10]. This reaction system can be applied to the detection of BGs using body fluids obtained from silkworm larvae [11,12]. We created a BG-binding protein, S-BGRP, by genetic modification of the insect BGRP [13]. In the design of the mutant, we modified 23 amino acids of the lepidopteran BGRP, while preserving the amino acid residues essential for BG binding [11]. Then, we could obtain the recombinant S-BGRP in high yield [13]. Furthermore, S-BGRP is a very stable protein that does not lose BG-binding ability even when heated to 90 °C [7]. Furthermore, S-BGRP maintains BG-binding ability over a wide pH range and exhibits more stable binding than antibodies [7,14].

Furthermore, we created a (1→6)-β-glucanase mutant protein (16BGM) with high specificity for fungal 1,6-β-glucan by replacing catalytic glutamic acid residue with glutamine to eliminate its hydrolase activity [15]. We have reported that 16BGM exhibits reactivity to various *Candida* fungi [16]. Since most of the fungal BGs have (1→3)-β-D-glucan and (1→6)-β-D-glucan, the combination of S-BGRP and 16BGM will enable the detection of fungal BGs with high specificity [17]. In this study, we developed a prototype BG reactive probe using S-BGRP and 16BGM and employed the scanning single-molecule counting (SSMC) method for detection.

## 2. Results

### 2.1. SSMC Method for BG Detection Using 16BGM and S-BGRP

**Figure 1:**
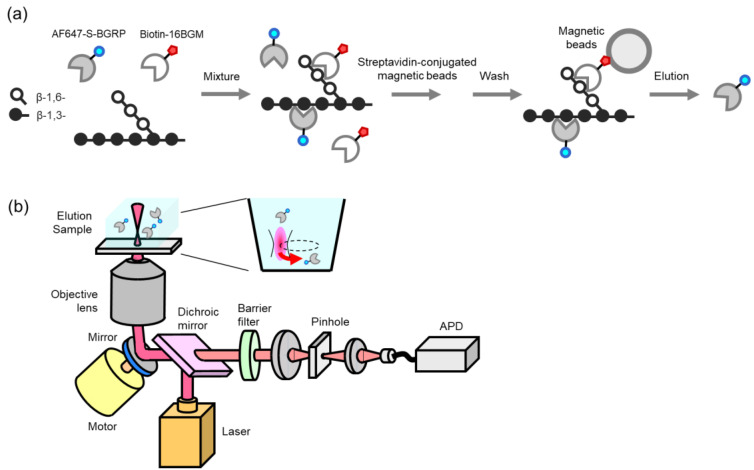
Schematic diagrams of molecular counting interacted with β-D-glucans. AF647-labeled molecules bound to BG complex were eluted (**a**), the elution sample was scanned, and a molecule in the confocal site was counted (**b**).

To investigate the BG reactivity of 16BGM and S-BGRP, we prepared biotin-labeled 16BGM and Alexa Fluor 647 (AF647)-labeled S-BGRP and examined them. Streptavidin-conjugated magnetic beads were mixed with both proteins and samples to dissociate the AF647-labeled S-BGRP from the samples captured by the beads, and the number of AF647 molecules in the elution was counted (Figure 1).

### 2.2. Comparison of BG Detection by LAL and SSMC

**Figure 2:**
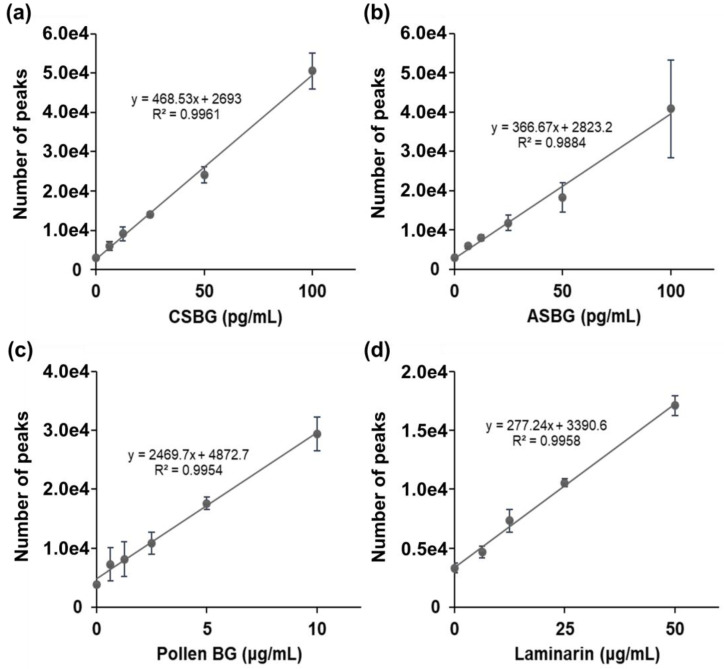
Detection of various BGs using S-BGRP and 16BGM. CSBG (**a**), ASBG (**b**), Pollen BG (**c**), and laminarin (**d**) are detected using 0.5 μg/mL AF647-labeled S-BGRP and 0.1 μg/mL biotin-labeled 16BGM. Each value represents a mean ± standard deviation (SD). *n* = 6 for 0 g/mL; *n* = 3 for all other concentrations.

Japanese cedar pollen is suggested to contain BG, which can be a false-positive for the diagnosis of invasive fungal infections [7]. Therefore, we also examined the SSMC method in combination with 16BGM and S-BGRP. First, we calculated the Pachyman equivalents of CSBG, ASBG, cedar pollen, and algae BG (laminarin) in 10 ng/mL of BG using the LAL method. As a result, 490 and 819 pg/mL of ASBG and CSBG, 51.6 pg/mL of pollen BG (Pollen BG) of Japanese cedar and 338 pg/mL of laminarin were found, as shown in Table 1. The fluorescence counts of CSBG, ASBG, Pollen BG, and laminarin at each concentration were determined using the SSMC method (Figure 2). The results showed that CSBG and ASBG had similar counts of approximately 5 × 10^4^ at 100 pg/mL, whereas the Pollen BG and laminarin count was lower, at approximately 3 × 10^4^ at 10 µg/mL and 1.7 × 10^4^ at 50 µg/mL, respectively, indicating that it is difficult to detect Japanese cedar pollen BG and algae BG using the SSMC method.

### 2.3. Correlation between LAL Method and SSMC Method

**Figure 3:**
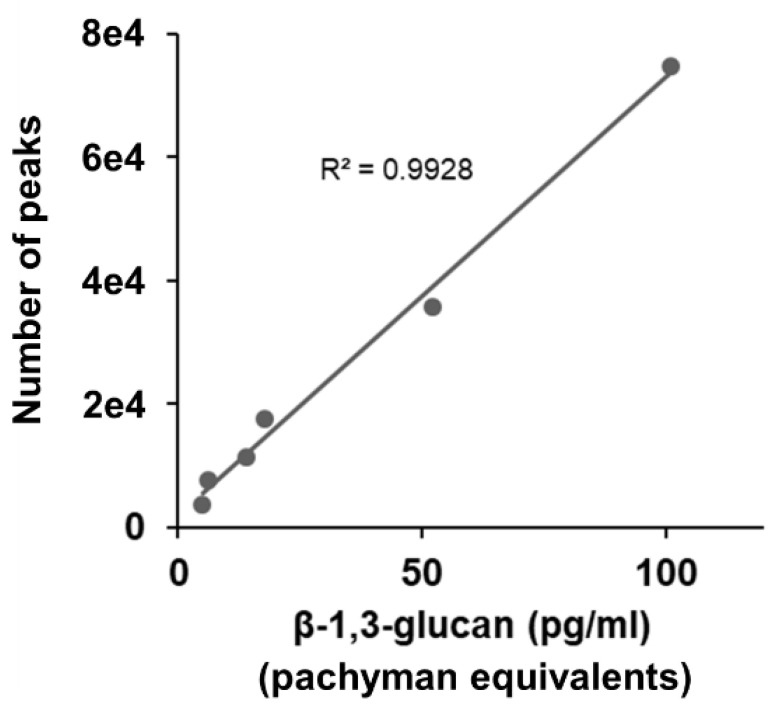
Comparison of LAL test and SSMC. 0–1000 pg/mL CSBG (two-fold serial dilution) are detected using LAL test and SSMC. A total of 0.5 μg/mL AF647-labeled S-BGRP and 0.1 μg/mL biotin-labeled 16BGM were used in SSMC.

As shown in Figure 3, there was a positive correlation between the Pachyman equivalent value of CSBG and the fluorescence count value of the SSMC method (Pearson’s R^2^ = 0.9928). These results indicate that the SSMC method has comparable reactivity to the LAL method for BG from candida.

### 2.4. Effect of Human Sera on the Reaction with CSBG in the SSMC Method

**Figure 4:**
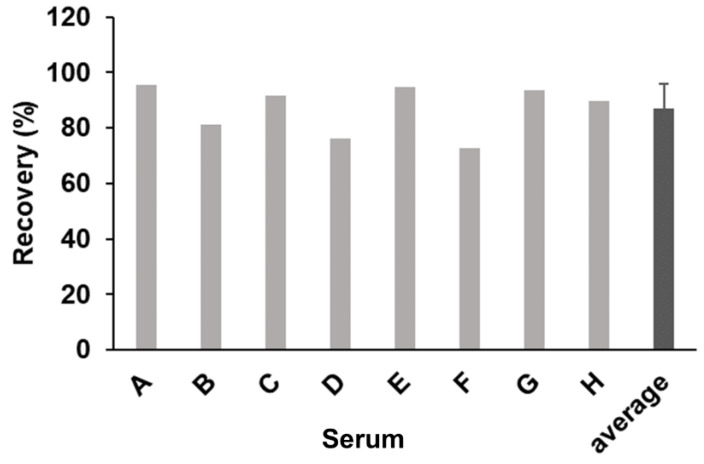
Spiking of CSBG into human sera and recovery test. A total of 50 pg/mL CSBG spiked into human sera were detected using 0.5 μg/mL AF647-labeled S-BGRP and 0.1 μg/mL biotin-labeled 16BGM. An error bar of average represents SD.

We then investigated the effect of human serum components on the detection of BG using the SSMC method with S-BGRP and 16BGM. We compared the detection of CSBG (50 pg/mL) in the presence of 10% human serum from eight different donors with that in the absence of serum (Figure 4). When CSBG measured in the absence of serum was set at 100%, the BG detection rates for all the normal human sera samples were between 73% and 95%, indicating that the presence of 10% serum did not significantly interfere with the detection of BGRP or the SSMC method using 16BGM.

### 2.5. Comparison of Reactivity to Immunoglobulin Preparations

Since it has been reported that some immunoglobulin preparations for intravenous injection may contain LAL-detectable BGs, we investigated the reactivity of the SSMC method to human immunoglobulin preparations [17]. Six immunoglobulin preparations were assayed by LAL and SSMC methods; the BG concentrations are shown as Pachyman and CSBG equivalents (Table 2). Venilon-I was low in both the LAL and SSMC methods, but Venoglobulin IH 5%, Gammagard, and Glovenin-I were above the detection limit in the LAL method. Polyglobin-N was detected as 60.6 and 41.1 pg/mL in the LAL and SSMC methods, respectively, whereas Sanglopor was detected as a high value of 1.5 ng/mL in the LAL method but was below the detection limit (32.4 pg/mL) in the SSMC method. These results showed that the SSMC method showed almost no positive results in the detection of BG in immunoglobulin preparations, whereas the LAL method registered high BG detection depending on the product.

## 3. Discussion

The mainstream serodiagnostic method for invasive fungal infection is the horseshoe crab Limulus assay [2,18], which is highly sensitive in detecting BGs but is also highly reactive to BGs other than fungi, resulting in problems in diagnostic accuracy.

Moro et al. reported the detection of BG in immunoglobulin preparations [19]. This report stated that several immunoglobulin products react positively in the LAL method and that these immunoglobulin products may be a source of false-positive substances [6,20]. In the present study, we also found that several immunoglobulin products contained LAL-reactive BGs. This outcome seems to be reproducible, as similar results have been obtained in other studies [20,21]. In contrast, the newly developed SSMC method for BG detection using a combination of 16BGM and S-BGRP did not detect BG in immunoglobulin products (Table 2) but showed high reactivity to candida-derived BG and CSBG. The reactivity against fungal BG was highly correlated with that of the LAL method, and the reaction remained effective even in the presence of serum components, indicating that the method could be applied to the detection of candida BGs in clinical specimens from infected patients. No clear answer has been found as to why Sanglopor showed LAL reactivity or what the substance was that reacted with the LAL method. Since our SSMC method suggests that (1→6)-branched (1→3)-β-D-glucan is specifically detected, the LAL-reactive substance in Sanglopor may contain only linear (1→3)-β-D-glucan. At least, it is unlikely that Sanglopor contains (1→6)-branched fungal-derived BGs.

Although certain cutoff values have been set for BG detection in the diagnosis of invasive fungal infection [22], the criteria for diagnosis in all cases are to detect BG at a concentration of several tens of pg/mL or higher [23]. Since (1→3)-β-D-glucan levels in the blood diagnostics of patients with fungal infections can be very low, at the pg/mL level, high sensitivity is required for diagnosis [2]. However, (1→3)-β-D-glucan is present not only in fungi but also in algae and higher plants [24,25,26]. Most fungal (1→3)-β-D-glucans can be structurally characterized by their branched (1→6)-β-glucan structure, and (1→6)-branched (1→3)-β-D-glucan is abundant in the cell walls of yeast and fungi [9]. In contrast, plant β-glucans contain 1,4-β-glucosyl-linked (1→3)-β-D-glucans, which are different from fungal (1→3)-β-D-glucans. Focusing on these structural features, we measured (1→6)-β-glucan and (1→3)-β-D-glucan to distinguish them from plant cellulose. Another approach for the simultaneous measurement of (1→6)-β-glucan and (1→3)-β-D-glucan can be performed using 16BGM and S-BGRP conjugated with fragment-luciferase, where both proteins accumulate on β-glucan and luciferase activity is expressed [17]. However, the BG concentration that can be detected by the fragment-luciferase-based assay is in the µg/mL range, and the assay lacks sufficient sensitivity for BG concentrations in the pg/mL range [17].

Several papers have been published on the detection of BGs by enzyme-linked immunosorbent assay. Using antibodies specific for (1→3)-β-D-glucan, it is possible to detect fungal BGs in the ng/mL range with a monoclonal antibody against laminarin and bovine serum albumin conjugate [27] and in the pg/mL range with a monoclonal antibody against laminariheptaose-human transferrin-conjugate antigen [28]. All of these are specific to the linear (1→3)-β-D-glucan structure, and the possibility of cross-reactivity with plant BGs other than fungi cannot be completely excluded [29].

The SSMC method is an ultra-sensitive analytical method with high sensitivity and a good signal-to-noise ratio, which can detect fluorescent molecules even at concentrations of several tens of aM [30]. To improve the measurement efficiency, we labeled the binding protein with the fluorescent dye Alexa Fluor 647, which is resistant to photobleaching [31], and adopted a method to rapidly count fluorescent molecules in a limited volume of the confocal site. The SSMC method has also been applied to the sensitive detection of oligonucleotide interactions hybridized to specific sequences [30]. The SSMC method has the potential to become an analytical method with excellent specificity and sensitivity for the detection of interactions between macromolecules and small molecules.

In this study, we successfully detected the interaction between polysaccharides and proteins with high sensitivity using the SSMC method. BG detection by the SSMC method uses recombinant proteins expressed in *E. coli* and is, therefore, inexpensive due to the availability of large quantities of reagents. This method has the potential to become a new diagnostic method for invasive fungal infections that can compensate for defects in the LAL method. Our next aim is to evaluate the practicality of this method in clinical specimens from patients with invasive fungal infections. In addition, since this measurement device is based on a confocal microscope system, one of the future tasks is to make the measurement device more compact. If a space-saving and compact measurement device can be realized, it may be used as a bedside measurement device.

## 4. Materials and Methods

### 4.1. Fungal Culture

*Aspergillus fumigatus* NBRC 30870 and *Candida albicans* NBRC 1385 were purchased from the National Institute of Technology and Evaluation (Tokyo, Japan). Fungi were maintained on Sabouraud agar (Difco, Franklin Lakes, NJ, USA) at 25 °C and transferred once every three months. To obtain yeast cells of *Candida albicans*, a C-limiting medium originally described by Shepherd and Sullivan [32] was used unless stated otherwise. The medium contained (per liter): sucrose, 10 g; (NH_4_)_2_SO_4_, 2 g; KH_2_PO_4_, 2 g; CaCl_2_·2H_2_O, 0.05 g; MgSO_4_·7H_2_O, 0.05 g; ZnSO_4_·7H_2_O, 1 mg; CuSO_4_·5H_2_O, 1 mg; FeSO_4_·7H_2_O, 0.01 g; biotin, 25 µg; final pH, 5.2. Five liters of medium were placed in the glass jar of a microfermenter (Sakura Seiki, Tokyo, Japan) and cultured at 27 °C with 5 L/min of aeration and stirring at 400 rpm. The cells were precipitated by adding ethanol and then dried with acetone.

### 4.2. Reagents

Sodium hypochlorite solution, sodium hydroxide (NaOH), and Dulbecco’s phosphate-buffered saline (PBS) were purchased from Wako Pure Chemical Industries, Ltd. (Tokyo, Japan). Distilled water (DIW) was purchased from Otsuka Co., Ltd. (Tokyo, Japan). Laminarin was purchased from Sigma-Aldrich (St. Louis, MO, USA). Human sera were purchased from BioIVT (Hicksville, NY, USA). Intravenous immunoglobulin products, Venilon-I, Venoglobulin IH 5%, Gammagard, Glovenin-I, Polyglobin-N, and Sanglopor were obtained from KM Biologics (Kumamoto, Japan), Japan Blood Products Organization (Tokyo, Japan), Shire Japan (Tokyo, Japan), Nihon Pharmaceutical (Tokyo, Japan), Japanese Red Cross Society (Tokyo, Japan), and CSL Behring (King of Prussia, PA, USA).

### 4.3. Preparation of Candida Albicans-Derived Soluble Beta-Glucan

Preparation of the NaClO-oxidized yeast was followed by the procedure used in a previous paper [33]. Briefly, *Candida albicans* NBRC 1385 yeast cells (2 g) were suspended in 200 mL of 0.1 M NaOH and oxidized with an appropriate volume of NaClO solution for 1 d at 4 °C. After the reaction was completed, the reaction mixture was dialyzed extensively with distilled water to collect the non-dialyzable and insoluble fraction, or the reaction product was directly centrifuged to collect the insoluble fraction. The insoluble fractions were dried by washing with ethanol and acetone. Each dried fraction was suspended in Me_2_SO and extracted by occasional sonication and boiling. After centrifugation to remove any insoluble fraction, the solubilized part was again precipitated with ethanol and acetone. The resulting Me_2_SO-soluble material was designated as CSBG.

### 4.4. Preparation of Aspergillus Cell Wall Glucans

Acetone-dried mycelium of Aspergillus fumigatus (2 g) was suspended in 200 mL of 0.1 M NaOH with NaClO at various available chlorine concentrations for 1 d at 4 °C. After the reaction was complete, the reaction mixture was centrifuged to collect the insoluble fraction. The insoluble fractions were dried by washing with ethanol and acetone (NaClO-treated Aspergillus, OX-Asp). OX-Asp suspended in 8 M urea was autoclaved at 120 °C for 20 min, and the resulting solutions were centrifuged (12,000 rpm, 20 min) and divided into supernatant and precipitant. Each fraction was dried in ethanol and acetone. The supernatant fraction was designated as ASBG.

### 4.5. Purification of Pollen BG Using BGRP-Assisted Affinity Chromatography

Preparation of the Pollen BG was followed by the procedure used in a previous paper [7]. To prepare the BGRP column, a Hitrap NHS-activated column (Cytiva, Marlborough, MA, USA) was conjugated with S-BGRP according to the manufacturer’s protocol. Before use for BG purification, the BGRP column was washed with 10 mL of PBS that was flowed through a peristaltic pump set at 2 mL/min. The pollen extract was prepared as follows. Five grams of Japanese cedar pollen (Fujifilm Wako, Osaka, Japan) were suspended in 1 L of 0.1M NaHCO_3_ aqueous solution and stirred for 30 min at room temperature. Then the supernatant was collected by 2 step centrifugations with 6000 × *g* for 10 min and 10,000 × *g* for 10 min. Finally, the supernatant was filtered with a 0.20 µm aPES bottle top filter (Thermofisher Scientific, Waltham, MA, USA). Then, 900 mL of the pollen extract was passed through the column at a rate of 2 mL/min. After washing the column with 10 mL of PBS (wash fluid), the BG was eluted as five fractions (900 µL/fraction) using 0.03 M NaOH. The eluates containing Pollen BG were immediately neutralized with 300 µL of 0.1 M phosphate citrate buffer (pH 3.0), dialyzed against deionized water, and lyophilized.

### 4.6. Preparation of S-BGRP and 16BGM

S-BGRP and 16BGM were prepared as recombinant proteins using the *E. coli* BL21 and pCold-I expression systems [15,16]. The proteins were purified using a TALON affinity resin column (Takara Bio, Shiga, Japan) as reported previously. The purified proteins were fluorescently labeled using the Alexa Fluor 647 Antibody Labeling Kit (Invitrogen, Carlsbad, CA, USA), and biotin-labeled using the Biotin Labeling Kit-NH_2_ (DOJINDO Laboratories, Kumamoto, Japan).

### 4.7. Basic Assay Method for SSMC

Ten microliters of β-glucan sample, 10 µL of human serum, and 60 µL of PBS containing 1% BSA were pretreated at 95 °C for 1 min and then mixed with AF647-labeled S-BGRP (5 µg/mL, 10 µL) and biotin-labeled 16BGM (1 µg/mL, 10 µL), and incubated at 37 °C for 30 min with agitation and light shielding. Then, 10 µg of streptavidin-conjugated magnetic beads (Invitrogen, Carlsbad, CA, USA) were mixed with the mixture and incubated at 37 °C for 1 min with agitation. The beads were washed five times with 100 µL of PBS containing 0.1% Triton X-100 and once with 10 mM glycine-HCl buffer (pH 2.5) containing 0.1% Triton X-100, and then 20 µL of 10 mM Tris-HCl (pH 8.0) containing 0.1% SDS was added to the magnetic beads. After incubation at 95 °C for 30 s, the filtrate containing AF647-labeled S-BGRP was eluted by filtering the supernatant with a 0.22 μm filter plate (Millipore, MA, USA) and measured by SSMC.

### 4.8. SSMC

SSMC used a laser light source (Showa Optronics, Tokyo, Japan) with a wavelength of 642 nm, barrier filter transmitting 660–710 nm (Chroma Technology, Bellows Falls, VT, USA), 40× water immersion objective lens (UAPON40XW340, NA = 1.15; Olympus, Tokyo, Japan), and avalanche photodiode (APD) (Perkin Elmer, Waltham, MA, USA). Optical scanning was performed at a scanning speed of 69 mm sec^-1^ and with an excitation light of 1 mW. Fluorescence time series data were acquired for 600 s and analyzed as previously described [30].

## Figures and Tables

**Table 1:** BG content of CSBG, ASBG, Pollen BG, and laminarin in the LAL test.

BGs (10 ng/mL)	LAL (pg/mL) (Pachyman Equivalents)
CSBG	490 ± 48
ASBG	819 ± 17
Pollen BG	51.6 ± 0.9
Laminarin	338 ± 2

BG content in the various BG preparations was determined by factor G-based LAL assay as a Pachyman equivalent in the clinical laboratory of SRL, Inc. Each value represents a mean ± standard deviation (SD) of three measurements.

**Table 2:** Comparison of LAL and SSMC methods for determination of BG content in intravenous immunoglobulin.

Intravenous Immunoglobulin (IVIG)	LAL (pg/mL) (Pachyman Equivalents)	SSMC (pg/mL) (CSBG Equivalents)
Venilon-I	<5.0	<32.4
Venoglobulin IH 5%	48.4	<32.4
Gammagard	36.8	<32.4
Glovenin-I	73.9	<32.4
Polyglobin-N	60.6	41.1
Sanglopor	1570	<32.4

BG content in the various immunoglobulin medicine products was determined by factor G-based LAL assay as a Pachyman equivalent in the clinical laboratory of SRL, Inc., and by SSMC as a CSBG equivalent, as described in Materials and Methods.

## Data Availability

Not applicable.
